# Recurrent Ectopic Pregnancy in the Tubal Remnant after Salpingectomy

**DOI:** 10.1155/2013/753269

**Published:** 2013-09-18

**Authors:** Bahareh Samiei-Sarir, Christopher Diehm

**Affiliations:** Western Sydney Local Health District, Australia

## Abstract

We present two cases of ectopic pregnancy located within the remnant tube following ipsilateral salpingectomy. This particular pathology is rare and yet has significant consequences for the patient, with mortality rates 10–15 times higher than other ectopic pregnancies. It demonstrates that salpingectomy does not exclude ectopic pregnancy on the ipsilateral side. We suggest careful clinical consideration and bring attention to the current surgical technique.

## 1. Introduction

Ectopic pregnancy occurs in around 1-2% of all pregnancies [1]. Ectopic pregnancy is still the most common cause of first trimester maternal death, accounting for 73% of early pregnancy mortality [2]. The incidence of ectopic pregnancy has increased markedly over the last three decades [3]. This is probably due to multiple factors such as the increased prevalence of pelvic inflammatory disease (PID), use of assisted reproductive technology [4], and increasing maternal age [5]. Ipsilateral ectopic pregnancy following salpingectomy (total or partial) is rare, with less than a dozen cases reported in the English literature in the last 10 years [6]. This particular type of ectopic pregnancy is associated with mortality rates 10–15 times higher than other ectopic pregnancies [7]. In this report, 2 cases of spontaneous ectopic pregnancy located in the remnant tube after ipsilateral salpingectomy are presented. 

## 2. Case Reports

### 2.1. Case 1

**Figure 1:**
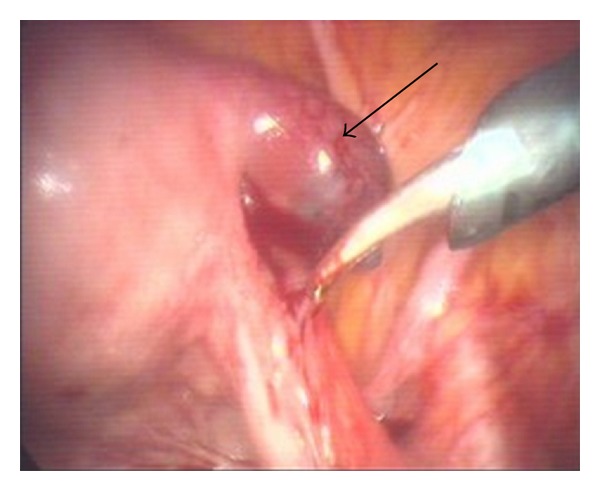
Ectopic pregnancy in remnant of right rube (arrow).

A 42-year-old female gravid 11, para 7 with 7 normal vaginal deliveries, 2 previous ectopic pregnancies, and one miscarriage. A right-sided ectopic was managed via salpingectomy in 2002. A left-sided ectopic resolved with expectant management in 2007. With this 3rd case of ectopic pregnancy the patient conceived spontaneously. No intra- or extrauterine gestation was visualised on 2 transvaginal ultrasounds in the preceeding week. Her serum *β*-HCG was monitored every 48 hrs during the preceeding week, which demonstrated levels that increased, but did not double (19,000 to 25,000 in 7 days). The patient presented to the Emergency Department with severe RIF pain and rebound tenderness. Laparoscopy revealed a large, bleeding, right ectopic pregnancy within the remnant of the right tube (see Figure 1), with a haemoperitoneum of approximately 500 mL. The ectopic was removed intact with monopolar/bipolar diathermy plus tubal ligation on the left side.

### 2.2. Case 2

**Figure 2:**
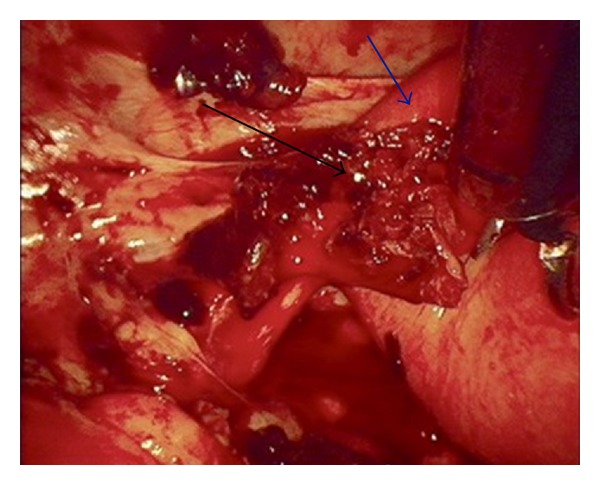
Ruptured ectopic pregnancy (black arrow) in remnant of left tube (blue arrow).

A 35-year-old female gravid 8, para 2, with 2 previous caesarean sections, 3 miscarriages, 1 termination of pregnancy, and 1 ectopic pregnancy on the left (salpingectomy, 2008). She conceived spontaneously and was at 49 days of gestation when she presented to the emergency department. The patient was tachycardic with abdominal guarding and rebound tenderness. Ultrasound demonstrated a left adnexal mass (4.3 × 2.6 mm) that showed features of a fetal pole and yolk sac plus free fluid in the Pouch of Douglas. *β*-HCG was 6500 IU/L. The patient was administered prophylactic anti-D and was transfused with 3 units of packed red cells (Hb 99 after transfusion). Laparoscopic intervention demonstrated a ruptured left ectopic pregnancy (see Figure 2) and a haemoperitoneum of approximately 2 L. Laparoscope also revealed multiple adhesions consistent with previous severe PID. 

## 3. Discussion

Approximately 92% of ectopic pregnancies occur in the ampullary region of the fallopian tubes, 2.5% as interstitial/cornual ectopic pregnancies, while less-common forms include cervical, ovary, and peritoneal [8]. Ectopic pregnancy occurring in the isthmic portion of the remnant tube following salpingectomy would be assumed to be even less common—especially following spontaneous conception. The exact incidence of ectopic pregnancy in the remnant stump following salpingectomy is not currently known. Takeda et al. reported an incidence of 1.16% in their department from January 1994 to August 2005 [8]. 

Isthmic ectopic pregnancy is a gynaecological emergency, with mortality rates around 2.0–2.5%. This contrasts with other ectopic pregnancies with mortality rates of around 0.14% [7]. This location is associated with high risk of rupture and severe bleeding at an early gestational age. This is due to the poor ability of this portion of the tube to distend as well as the increased vascularity of the area (anastomosis of the uterine and ovarian vessels) [8]. This situation is evident in case number two, whereby the patient presented at 7-weeks gestation with an acute abdomen. She had haemoperitoneum of 2 litres and required transfusion with 3 units of blood. 

The mechanism by which ectopic pregnancy in the remnant tube after salpingectomy occurs is not clear. Reported hypotheses include Spermatozoa pass through the patent tube, into the Pouch of Douglas, and travel to fertilise the ovum on the side of the damaged tube [4]. An oocyte from the left ovary may be fertilised normally in the patent tube and then later implant in the stump via intrauterine migration [8]. Another possibility is that, despite the ligation of the tube following salpingectomy, some degree of patency or recanalisation may occur. This thus provides a communication between the endometrial and peritoneal cavities and allows for fertilisation and implantation within the isthmic portion of the remnant tube [9]. 

Given the uncertain nature of the mechanism, selecting a method for prevention is difficult. However, a few options may be suggested to decrease the probability of recurrence. When performing the salpingectomy, care should be taken not to leave a long stump remaining [4]. It should be noted that, generally, it is common practice to leave a long tubal stump to minimise the risk of bleeding associated with the isthmic portion of the fallopian tube [8]. However, given the risk of future ectopic pregnancies in those with a history of ectopic pregnancy, it may be suggested that this remnant portion should be minimised. Additionally, adequate diathermy of the proximal portion or ligation with clips may be necessary components to decrease the risk of future implantation. 

Another suggestion in management includes performing Hysterosalpingography to evaluate the patency of the fallopian tubes after salpingectomy and ligation. In addition to salpingectomy, one of the authors suggests the insertion of flexible microinserts (commercial products are available) into the remnant tube. These devices are generally considered to be effective in occluding the fallopian tubes [10]. This may provide greater protection through more definitive occlusion of the proximal tube. Alternatively, if the woman has completed her family and has a history of ectopic pregnancy, effective contraception counseling may be given, or permanent contraceptive measures implemented. 

## 4. Conclusion

The rate or occurrence of this type of ectopic pregnancy is not known; however, a small sample suggests 1.16% of all ectopic pregnancies with mortality 10–15 times higher compared to other forms of ectopic. Clinicians should be aware that one ectopic is a risk factor for future ectopics and that salpingectomy does not exclude ipsilateral ectopic pregnancy. When performing a salpingectomy, we suggest that the length of the remnant should be minimised and adequate diathermy applied. Finally, assessment of remnant stump patency (via hysterosalpingography) should be considered and tubal occlusion devices may be used to interrupt any remaining patency.
